# Contemporary management of rectal cancer

**DOI:** 10.1016/j.sopen.2024.01.009

**Published:** 2024-01-19

**Authors:** Alexander M. Troester, Wolfgang B. Gaertner

**Affiliations:** a Department of Surgery, University of Minnesota, Minneapolis, MN, United States of America; b Division of Colon & Rectal Surgery, Department of Surgery, University of Minnesota, Minneapolis, MN, United States of America

**Keywords:** Rectal cancer, Total neoadjuvant therapy, Endorectal ultrasound, Pelvic lymph node dissection, Short-course radiotherapy

## Abstract

The management of rectal cancer has undergone significant changes over the past 50 years, and this has been associated with major improvements in overall outcomes and quality of life. From standardization of total mesorectal excision to refinements in radiation delivery and shifting of chemoradiotherapy treatment to favor a neoadjuvant approach, as well as the development of targeted chemotherapeutics, these management strategies have continually aimed to achieve locoregional and systemic control while limiting adverse effects and enhance overall survival. This article highlights evolving aspects of rectal cancer therapy including improved staging modalities, total neoadjuvant therapy, the role of short-course and more selective radiotherapy strategies, as well as organ preservation. We also discuss the evolving role of minimally invasive surgery and comment on lateral pelvic lymph node dissection.

**Key message:**

Rectal cancer management is constantly evolving through refinements in radiation timing and delivery, modification of chemoradiotherapy treatment schedules, and increasing utilization of minimally invasive surgical techniques and organ preservation strategies. This manuscript aims to provide a synopsis of recent changes in the management of rectal cancer, highlighting contemporary modifications in neoadjuvant approaches and surgical management to enhance the knowledge of surgeons who care for this challenging population.

## Introduction

While rectal cancer has been a recognized pathology for millennia, it wasn't until 1739 when Jean Faget of France first attempted rectal resection [1]. Nearly a century later, in 1826, Jacques LisFranc was credited with the first successful excision of a rectal tumor [1]. The “radical abdominoperineal resection” (APR), first described by Sir William Ernest Miles in a 1908 *Lancet* article [2], and became commonplace by 1938. With the advent of blood transfusion and improved anesthetic after World War II, operative morbidity and mortality from radical resection was reduced and surgeons began exploring more sphincter-preserving procedures [[3], [4], [5]]. In the 1980s, Richard Heald coined the term “total mesorectal excision” (TME) when he excised a rectal tumor and mesorectum *en bloc* to the level of levator muscles sharply under direct vision [6]. Guided by Heald's anatomic principles, TME remains the predominant method for surgical resection of rectal cancer today.

Paralleling advances in surgical technique for rectal cancer, delivery of chemotherapy and radiation have evolved over the past century. Studies as early as the 1960's described the benefits of preoperative radiation therapy in patients with locally advanced rectal cancer (LARC) [7,8]. Yet, well into the 1990's, post-operative radiation therapy combined with chemotherapy was the standard of care [9]. Over the past few decades, therapy-related toxicities and poor treatment tolerance have led to trials comparing pre-operative and post-operative therapies. Multiple large trials have demonstrated the benefit of pre-operative radiotherapy (RT) combined with chemotherapy [[10], [11], [12]], making neoadjuvant chemoradiotherapy (CRT) the current standard of care for LARC.

Rectal cancer management has improved immensely over the past 50 years and continues to evolve. The aim of this review is to highlight recently investigated aspects of rectal cancer treatment including short-course (SCRT) and more selective radiotherapy as components of total neoadjuvant therapy (TNT) focusing on the RAPIDO, OPRA, and PROSPECT trials; the role of organ preservation with a clinical complete response (cCR), and finally contemporary data on the minimally invasive surgical (MIS) approach to rectal cancer with a comment on the utility of lateral pelvic lymph node dissection (LPLND), especially in the setting of evolving neoadjuvant strategies.

## Total neoadjuvant therapy

Neoadjuvant long-course CRT has been the standard of care for LARC for many years in the United States. This was typically followed by TME and adjuvant systemic chemotherapy with good locoregional control and widely reported low local recurrence rates. Despite these improvements, there was an increasing incidence of distant metastatic disease in up to 25 % in patients with LARC treated with this approach [[13], [14], [15]]. At the same time, prospective data on the benefits of waiting longer time intervals (8–10 weeks) between the completion of CRT and TME, as well as delayed delivery of adjuvant systemic chemotherapy with frequent interruptions because of postoperative and stoma-related complications [16]. This time interval between CRT and TME was seen as an opportunity to safely deliver systemic chemotherapy in order to assure timely delivery of systemic therapy with the potential to reduce the risk of systemic failure and secondarily to shorten the time interval to potential loop ileostomy reversal, when performed [17,18]. Thus, shifting delivery of systemic chemotherapy into the neoadjuvant period along with CRT comprised the idea of total neoadjuvant therapy (TNT).

The EXPERT and EXPERT-C trials evaluated 269 high-risk tumors in patients receiving oxaliplatin-based neoadjuvant chemotherapy plus CRT, showing excellent compliance to chemotherapy (91 %) and CRT (88 %), with a 73 % 5-year overall survival [[19], [20], [21]]. Similarly, the Spanish GCR-3 trial demonstrated significantly higher rates of chemotherapy completion when given in the neoadjuvant period (91 % *vs.* 51 %; *P* < 0.01) along with less grade 3+ toxicity (19 % *vs.* 54 %; P < 0.01) [22]. Superior tolerability, in conjunction with an opportunity to achieve a cCR, defined as the absence of residual tumor after nonoperative therapy determined by physical, radiologic, and endoscopic evaluation, led to more adoption of a watch and wait (WW) approach. This is how TNT started and beginning in 2018, the National Comprehensive Cancer Network (NCCN) guidelines included TNT as a treatment option for LARC [23]. Subsequent refinements in the delivery of TNT have largely evaluated timing of systemic chemotherapy either before CRT (induction chemotherapy) or after (consolidation chemotherapy) and its effects both on tumor response rates [24] and organ preservation [25].

### Short-course radiotherapy

As a component of LARC treatment, two main forms of radiation courses have been used based on duration: short-course *versus* long-course chemoradiotherapy (LCRT). Historically, the rationale for LCRT included reducing the risk of local recurrence by concurrently causing tumor downstaging with the addition of radiosensitizing chemotherapy thereby potentially increasing the chance of R0 TME [12]. Meanwhile SCRT, given as five fractions of five Gray radiation (5 × 5 Gy) over the course of one week, was traditionally used to reduce the risk of local recurrence in intermediate-risk resectable rectal cancer because downsizing of the tumor was not required. While SCRT was associated with less toxicity compared to LCRT, concerns remained regarding the use of high radiation doses per fraction and its potential impact on postoperative healing and late toxicity [11,12,26]. Due to these concerns, two randomized controlled trials (RCT) investigated SCRT *versus* LCRT and found no significant differences in oncologic or late toxicity outcomes [26,27].

In order to further explore the potential benefits of SCRT, the Swedish Rectal Cancer Trial randomly assigned patients to non-TME surgery alone *versus* SCRT + non-TME surgery, showing improved overall survival (30 % *vs.* 38 %, *P* = 0.008) and decreased local recurrence rates (26 % *vs.* 9 %, *P* < 0.001) in the SCRT + surgery group [28]. Long-term results from the Dutch TME trial comparing TME *versus* SCRT + TME demonstrated a reduction in the 10-year cumulative incidence of local recurrence (11 % *vs.* 5 %, *P* < 0.001) with addition of SCRT [29]. Further, the Stockholm III trial expanded SCRT applicability showing that radical resection rates and postoperative complication risks were not compromised by delaying surgery for 4–8 weeks after SCRT [30,31]. In addition, oncologic outcomes after SCRT and delayed surgery were not inferior to LCRT [31]. What remained unclear after these trials was how neoadjuvant SCRT and LCRT compared in patients with locally advanced rectal cancer as only the Dutch trial performed TME. In addition, there were unresolved caveats such as suboptimal imaging staging with inherent inconclusive data on threatened circumferential margin status and mesorectal lymphadenopathy.

### RAPIDO trial

With retrospective single-institution series showing that TNT was safe and well tolerated, associated with higher cCR and pathologic complete response (pCR) rates, and not associated with increased postoperative complications, and knowing that in high-risk rectal cancers (T4, N2, extramural vascular invasion, involved mesorectal fascia, or enlarged lateral pelvic lymph nodes), compliance to adjuvant chemotherapy was poor [[32], [33], [34]], the Rectal cancer And Preoperative Induction therapy followed by Dedicated Operation (RAPIDO) trial compared SCRT followed by systemic chemotherapy *versus* LCRT before TME as a means of achieving higher compliance, downstaging, and better effect of systemic therapy [35,36]. Patients were randomized to receive either SCRT followed by systemic chemotherapy with TME 3–14 days later as the experimental group (EXP) or LCRT with concomitant oral chemotherapy followed by TME after a 6–10 week waiting period +/− adjuvant chemotherapy as the standard group (STD) [35]. Of the 450 eligible patients in the STD group, only 187 (42 %) completed adjuvant chemotherapy. Notably, pCR rates in the EXP arm were double the STD group (28 % *vs.* 14 %, *P* < 0.0001). At 3 years, the probability of disease-related treatment failure was improved in the EXP group although no difference was seen in the cumulative probability of locoregional failure [35].

A recent update, with a median follow up of 5.6 years, provided a long-term analysis again showing overall locoregional failure rates did not differ between EXP and STD groups [37]. However, of the 857 patients who underwent an R0 or R1 resection, there was a higher rate of locoregional recurrence in the EXP group (10.2 % *versus* 6.1 %, *P* = 0.03) with 9/44 (21 %) EXP patients who experienced a locoregional recurrence having a breached mesorectum on pathologic evaluation compared to just 1/26 (4 %) in the STD arm (*p* = 0.048). Oncologic outcomes at 5 years demonstrated a reduction in disease-related treatment failure in the EXP group as well as a decrease in cumulative probability of distant metastases. However, this did not translate into a 5-year overall survival benefit (EXP 81.7 % *vs.* STD 80.2 %, HR 0.91, 95 % CI 0.70–1.19) [37].

Results from the RAPIDO trial reveal a potential survival paradox where the EXP group had higher pCR rates that failed to translate into an OS benefit suggesting the effect may be secondary to a variety of additional factors. Furthermore, the intensification of neoadjuvant regimens could improve initial tumor response in patients whose biology remains at high risk of treatment failure. Surgical technique in the era of TNT remains a key tenant of comprehensive rectal cancer management to ensure that the mesorectum is intact on pathologic analysis, as the increased risk of locoregional recurrence in the EXP group was most pronounced in the breached group.

### OPRA trial

While the RAPIDO trial compared outcomes following TNT (SCRT plus consolidation chemotherapy) or LCRT followed by TME for all patients, the Organ Preservation for Rectal Adenocarcinoma (OPRA) trial incorporated a WW management approach for patients receiving TNT who achieved a near complete (nCR) or cCR [25]. Those with an incomplete response were recommended to undergo TME. The OPRA trial randomized patients to receive induction or consolidation chemotherapy and assessed effects at 3 years. Although survival outcomes did not differ between induction and consolidation chemotherapy arms, the proportion of patients who preserved their rectum was 60 % in the consolidation arm compared to 47 % in the induction arm (*p* = 0.02) [25].

The OPRA trial showed that organ preservation can be achieved in half of LARC patients treated with TNT, particularly when consolidation chemotherapy is utilized. However, the long-term outcomes from the OPRA trial are still pending. Nevertheless, physicians will need to have multidisciplinary discussions to balance the risk of disease recurrence alongside patient preference.

### PROSPECT trial

The recently published PROSPECT trial examined whether neoadjuvant chemotherapy using fluorouracil, leucovorin, and oxaliplatin (FOLFOX) with selective CRT was noninferior to standard neoadjuvant CRT in LARC patients amenable to sphincter-preserving surgery [38]. Selective chemoradiotherapy was indicated if the primary tumor did not decrease in size by at least 20 % after FOLFOX, or if FOLFOX was discontinued due to side effects, which occurred in 9.1 % of patients. Both groups had similar rates of pCR, and although not mandated in the pre-trial protocol, there were similar rates of adjuvant chemotherapy utilization (74.9 % *vs.* 77.9 %). At 5 years follow-up and with adjustment for age and radiologic nodal status, FOLFOX was noninferior to chemoradiotherapy in terms of DFS (80.8 % *vs.* 78.6 %, respectively), but the groups were similar with respect to OS and local recurrence (LR).

Clinician-reported grade 3+ adverse events during neoadjuvant therapy were higher in the FOLFOX group than the CRT cohort (41.0 % *vs.* 22.8 %), however this data should be interpreted with caution. The treatment period in the FOLFOX arm was a minimum of 12 weeks *vs.* only 5.5 weeks for standard of care arm. Additionally, adherence to ≥5 cycles of FOLFOX was 94.9 %, whereas only 68.7 % and 70.1 % of patients received full doses of neoadjuvant 5-FU and oxaliplatin, respectively. Conversely to neoadjuvant event rates, post-operative adverse events occurred with greater frequency in the CRT group, ultimately highlighting that toxic effects are associated with FOLFOX administration.

The PROSPECT trial demonstrated that a large proportion of carefully selected LARC patients can avoid pelvic radiation along with its short- and long-term sequelae, while not compromising pCR, DFS, OS, or LR rates. Long-term follow up of this cohort will be crucial to assess the magnitude of late effects. Nonetheless, the PROSPECT trial expanded the therapeutic options for carefully selected LARC patients.

## Importance of MRI in staging

Rectal cancer protocol pelvic magnetic resonance imaging (MRI) is the preferred modality for locoregional clinical staging according to current American Society of Colon & Rectal Surgeons (ASCRS) guidelines [39]. MRI staging of rectal cancer allows for the assessment of tumor penetration depth, topographic relationship of the tumor and nodal metastases to the mesorectal fascia, and detection of locoregional nodal metastases [40]. This information is crucial in predicting surgical clearance of the circumferential resection margin (CRM), defined as the closest distance of the tumor to the mesorectal fascia [[41], [42], [43]]. A positive CRM, defined as either cancer within 1 mm [43] or 2 mm [44] from mesorectal fascia and levator muscles and not invading into the intersphincteric plane, has been associated with increased risk of local recurrence and decreased survival [45,46].

In addition to a positive CRM, lymph node positivity has a negative impact on recurrence and survival rates [45,46]. Yet, detection of potentially involved mesorectal, lateral pelvic, and inguinal lymph nodes remain a diagnostic challenge. Historically, sensitivity (MRI 66 %; CT 55 %) and specificity (MRI 76 %; CT 74 %) of lateral lymph node metastases have been similar between imaging modalities [47]. A recent meta-analysis confirmed the difficulty of MRI detection of lateral lymph node metastases (pooled sensitivity 77 %, pooled specificity 71 %), while demonstrating its superiority of classifying T stage and evaluating the CRM [48]. Overlap between size of benign and malignant lymph nodes exists, and size criteria for prediction of metastatic nodes is highly variable [[49], [50], [51], [52], [53], [54], [55], [56], [57], [58]]. Moreover, micrometastases in small nodes can be easily missed. As MRI technology continues to advance, it may improve nodal staging accuracy by characterization of signal intensity relative to tumor and integration of morphologic criteria such as indistinct or spiculated borders [59,60]. Accurate preoperative determination of lymph node status not only informs prognostic discussions, but also decisions regarding choice of neoadjuvant treatment schedule and whether to perform lateral pelvic lymph node dissection (LPLND).

## Endorectal ultrasound: delineating T1 *vs* T2 lesions

Rectal cancer typically appears as a hypoechoic mass on endorectal ultrasound (ERUS) that disrupts the normal 5-layer sonographic structure of the rectal wall through tumor infiltration causing fusion of these layers [61,62]. Given that MRI cannot fully characterize early T1/T2 lesions, ERUS can be a valuable modality for these early tumors [47,61,63,64] and is also useful when MRI is contraindicated due to the presence of an implantable medical device. Determining depth of invasion is critical, as T1 lesions with favorable features (well to moderately differentiated, lack of lymphovascular or perineural invasion, low tumor budding, <30 % bowel circumference, and < 3 cm in size) may be offered local excision in the form of transanal excision, transanal endoscopic microsurgery (TEM) or transanal minimally invasive surgery (TAMIS) [23]. Compared to radical surgery, these approaches have been associated with reduced patient morbidity, improved anorectal functional outcomes compared to TEM even without CRT, and acceptable oncologic outcomes in carefully selected patients [65].

Even with the advantages of MRI in the staging of rectal cancer, there continues to be a role for ERUS in the staging of early rectal lesions. One previous meta-analysis found that ERUS and MRI had similar sensitivity of 94 %, but ERUS demonstrated a better specificity (94 % *versus* 86 %) in detection of muscularis propria invasion [47]. Kwok *et al* [66] had similar observations on the increased accuracy of ERUS but added that MRI technique using an endorectal coil was as effective as ERUS in assessing rectal wall penetration, although this MRI technique is no longer performed.

While routine use of ERUS in the evaluation of early-stage rectal cancer is supported by its T-staging accuracy, ERUS has several notable limitations. These include operator dependency, inability to evaluate stenotic tumors that preclude passage of the transducer, assessment of proximal rectal tumors, and failure to characterize lymph nodes that fall outside the range of the transducer [47,64,67]. Thus, although ERUS is a useful adjunct in the assessment of early tumor invasion depth, it does not supplant the routine evaluation of rectal cancer by MRI.

## Minimally invasive surgery approach to rectal cancer

Paralleling improvements in imaging modalities for rectal cancer over time, minimally invasive surgical approaches have been associated with improved short-term perioperative outcomes when compared to open surgery, with long-term oncologic results from randomized controlled trials demonstrating no significant differences [[68], [69], [70], [71]]. Although the ACOSOG Z6051 and ALaCaRT trials reported inferior pathologic outcomes (determined by a composite end point of CRM >1 mm, negative distal margin, and TME completeness) in the laparoscopic surgery arms compared to open surgery [72,73], long-term recurrence rates and survival outcomes have not been impacted [74,75]. Furthermore, as robotic rectal cancer surgery has continued to gain prominence, randomized controlled trials and meta-analyses have showed no differences in pathologic outcomes compared to laparoscopic surgery [[76], [77], [78]].

Low cancers, narrow pelvic anatomy, and high BMI are a non-comprehensive list of factors impacting the ability to complete a high-quality TME dissection laparoscopically, thus surgeons proposed the transanal TME (TaTME) approach to alleviate these shortcomings ultimately demonstrating similar perioperative and oncologic outcomes [79,80]. TaTME necessitates a low anastomosis potentially leading to suboptimal functional sequelae, along with risk of urethral injury and CO2 emboli rarely encountered in laparoscopic TME. Despite these potential limitations and lack of prospective, randomized data, TaTME remains a useful technique in the armamentarium of surgeons with the proper training with this technique who encounter low rectal cancers in patients with anatomic limitations.

## Lateral pelvic lymph node dissection

LPLND involves removal of the common iliac, internal iliac, and obturator nodal compartments. As mentioned previously, determination of lymph node positivity on pretreatment staging MRI remains a challenge, with a lack of consensus on predictive size criteria. Using a cutoff of >7 mm, the International Lateral Node Study Consortium found that patients who underwent CRT followed by TME + LPLND had reduced local recurrence when compared to patients who did not undergo LPLND (5.7 % *versus* 19.5 %, *P* = 0.04) [81]. The JCOG0212 trial, examining TME *versus* TME + LPLND in patients who did not undergo CRT, found that lateral pelvic node size >5 mm on initial staging MRI was associated with pathologically positive lateral nodes (OR 4.06; 95 % CI 1.59–10.24, *P* = 0.003) [82]. An *ad-hoc* analysis of the ACTS-RC trial demonstrated no advantage of LPLND on relapse-free survival (HR 0.94; 95 % CI 0.70–1.27) or OS (HR 0.86; 95 % CI 0.60–1.22) [83]. While some studies have demonstrated reduced local recurrence rates, studies have failed to show that LPLND confers a survival benefit, and is associated with significant postoperative functional sequelae, especially urinary dysfunction [84]. This is especially important with lack of data on lateral lymph node positivity in the setting of an increasing use of induction chemotherapy with selective CRT and TNT in general. LPLND safety and feasibility has been demonstrated through a minimally invasive approach [[85], [86], [87], [88]].

## Conclusions

In conclusion, contemporary management of rectal cancer has experienced vast improvements over the last 50 years due to delivery of targeted neoadjuvant chemoradiotherapy regimens, enhanced imaging modalities for accurate staging and ongoing surveillance, and refinement of surgical techniques utilizing a minimally invasive approach. As more knowledge is amassed regarding tumor biology and individualized treatment approaches, the complexity of rectal cancer management strategies will continue to improve. Imperative to the increasing complexity will be multidisciplinary discussions to select a management strategy that aims to maximize tumor response, limit recurrence, and improve survival all while maintaining a high quality of life.

## Funding sources

None.

## Ethical approval statement

Not required. This is a review article that did not access patient information.

## CRediT authorship contribution statement

**Alexander M. Troester:** Conceptualization, Data curation, Formal analysis, Writing – original draft, Writing – review & editing. **Wolfgang B. Gaertner:** Conceptualization, Supervision, Writing – review & editing.

## Declaration of competing interest

Dr. Gaertner has received proctor and speaker fees from Intuitive Surgical, advisory board and consultant fees from Coloplast, consultant fees from Applied Medical, and advisory board and consultant fees from Becton Dickinson. All other authors have nothing to disclose.
